# Distinct Effects of *Lactiplantibacillus plantarum* HNU082 on Microbial Single-Nucleotide Variants in Large Intestine and Small Intestine

**DOI:** 10.3390/microorganisms13040731

**Published:** 2025-03-25

**Authors:** Wenyao Ma, Zhe Han, Xinlei Liu, Weipeng Cui, Dongyu Zhen, Xiaolu Zhou, Yuan Song, Shuaiming Jiang

**Affiliations:** Key Laboratory of Food Nutrition and Functional Food of Hainan Province, School of Food Science and Engineering, Hainan University, Haikou 570228, China; mawenyao2000@163.com (W.M.); olaaaa1219@163.com (Z.H.); lxltracy2022@163.com (X.L.); cwpeng121996@163.com (W.C.); zhendy2001@163.com (D.Z.); zxl782072778@163.com (X.Z.); songyuan200085@126.com (Y.S.)

**Keywords:** probiotics, *Lactiplantibacillus plantarum* HNU082, gut microbiota, single-nucleotide variants

## Abstract

The intestinal tract extends several times the length of bodies, with varying environmental conditions across different segments (small intestinal and large intestinal), thereby harboring distinct gut microbiota. Most studies focused on the quantitative responses of gut microbiota upon probiotics entering the gut, without an in-depth analysis of how the genetic change in local gut microbiota. Therefore, in this experiment, C57BL/6J male mice were once administered *Lactiplantibacillus plantarum* HNU082 (Lp082). Then, the mice were euthanized on the 1st, 3rd, and 7th days after gavage, and the contents of the small and large intestines of the mice were scraped for metagenomic analysis. Based on the characterization of large intestine and small intestine bacteria, changes in the diversity and abundance of single-nucleotide variants (SNVs) of microbiota were analyzed. There were observable distinct responses at the genetic level. A significant number of SNVs were identified in *Ligilactobacillus murinus* in the large intestine. These SNVs may impact the utilization of carbohydrates in *L. murinus*. Ingested probiotics traversed the entire gut and interacted with the indigenous microbiota, driving the evolution of the indigenous gut microbiota in the different intestinal segments, thereby influencing microbial growth and metabolism. This study investigates the role of probiotics in the evolution of gut microbiota. It offers new probiotic insights and a basis for targeted interventions.

## 1. Introduction

With the progression of molecular biological technologies, a more profound investigation has been conducted into the metagenomics of gut microbiota [1]. Gut microbiota refers to the vast number of microorganisms residing in the animal gut, with bacteria accounting for 90% of these microorganisms [2]. These microorganisms play a crucial role in the host’s health status through processes such as nutrient absorption and metabolism. Additionally, they are involved in immune system regulation and intestinal barrier function modulation [3]. Gut microbiota regulates the host’s amino acid utilization by influencing intestinal proteolytic enzymes and altering intestinal permeability. It can also directly utilize or metabolize amino acids in the gut to maintain host health [4]. Gut microbiota plays a crucial role in host immune defense. It regulates the population of intestinal IgA-producing cells and modulates the expansion of specific lymphocyte subsets [5]. Gut microbiota can exert potential effects on brain function through the brain–gut microbiota (BGM) axis, either by activating the vagus nerve directly or indirectly by influencing the immune system [6].

The intestinal tract can extend to 4–5 times the length of the body, with distinct environmental conditions across different intestinal segments. These conditions include variations in epithelial cell types, mucus, pH, oxygen levels, intestinal structure, contractility, motility, and transit time of contents [3]. Additionally, immune factors also exert a certain influence on the species and abundance of microorganisms colonizing and functioning within the intestine [7,8]. The small intestine harbors the majority of intestinal receptors and constitutes an oxygenated environment. It is abundant with digestive enzymes, bile, intestinal fluids, and digestive contents, characterized by high fluidity and robust mechanical clearance. Consequently, the bacterial load in the small intestine is relatively low, ranging from 10^4^ to 10^5^ CFU/mL. The large intestine, comprising the cecum, colon, and rectum, serves as the primary site for the colonization and functional activity of intestinal microbiota. It is characterized by prolonged retention of intestinal contents, abundant nutrient supply, slower intestinal motility and digestive fluid flow, anaerobic conditions, and low redox potential. These various factors promote the colonization and proliferation of microbiota, ultimately leading to a highly diverse and densely populated microbial community. The large intestine bacterial count reaches 10^11^–10^12^ CFU/mL of intestinal contents, with a complex and varied species assemblage, which had anaerobic bacteria primarily including *Bacillota*, *Bacteroidetes*, *Actinobacteria*, and *Proteobacteria* [9].

Probiotics are live microorganisms that, when consumed in adequate amounts, confer health benefits to the host [10]. Probiotics can modify the gut microbiota structure, thus promoting the growth of beneficial bacteria and the production of beneficial metabolites [11]. After entering the human body, probiotics traverse the entire intestinal tract. They compete with the local microbiota in different intestinal segments for nutrients and ecological niches [12]. Consequently, probiotics cannot stably colonize the gut after a single gavage. Our previous study demonstrated that *Lactiplantibacillus plantarum* HNU082 (Lp082) could only be isolated from fecal samples within the first 7 days following a single administration [13]. Moreover, the competition between probiotics and the local microbiota also imposes selective pressure on the gut microbiota [14]. And brings intestinal selection pressure to the gut microbiota. Current research primarily focuses on the quantitative responses of gut microbiota, with inadequate in-depth analysis of the genetic alterations within the indigenous gut microbiota following probiotic intervention. Yet, the alterations at the genomic level within gut microbiota may influence the transcription, expression, and ultimately the functionality of these genes, with profound implications for the health of the host [15].

Therefore, this study intended to explore the genomic alterations in indigenous microbial populations across different intestinal segments in mice, which followed Lp082 intervention. Lp082 is a probiotic strain isolated from Yucha [10,14,16,17].

Under the intervention of Lp082, the indigenous microbiota will respond at a structural and genetic level. And the responses will be inconsistent across different intestinal segments. This result implies that future evaluations of probiotics need to consider the impact at the genetic level. It supplements the evaluation system for probiotics and delves deeper into the effects of probiotics on the microbiome. This study thereby enhances the mechanistic research on the efficacy evaluation of probiotics.

## 2. Methods and Materials

### 2.1. Preparation of Strains for Gavage

The cryopreserved Lp082 was inoculated into a sterilized MRS broth liquid medium and cultivated at 37 °C for 24 h. A 2 mL aliquot of the Lp082 culture, which had been incubated at 37 °C for 24 h, was thoroughly mixed and centrifuged at 5000× *g* for 5 min. The resulting pellet was resuspended in 200 μL of sterile physiological saline, which contained 10^10^ CFU of Lp082.

### 2.2. Experimental Design

C57BL/6J specific pathogen free (SPF) male mice were obtained from Beijing Vital River Laboratory Animal Technology Co., Ltd. (Beijing, China). These mice were individually housed in SPF-grade animal rooms maintained at 25 °C with 55% humidity, under a 12 h light/dark cycle. The bedding was changed every 3 days, and mice had free access to SPF-grade maintenance feed and distilled water. The experiment was reviewed and approved by the Ethics Committee of Hainan University (HNUAUCC-2023-00174).

**Figure 1:**
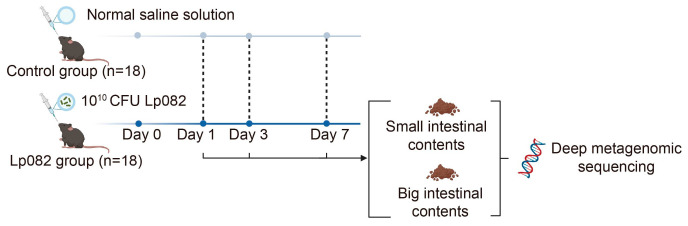
The experimental design flowchart.

Following seven days of adaptive feeding, the mice were randomly divided into two groups, 18 in the control group and 18 in the Lp082 group. The Lp082 group was administered a single gavage of Lp082 at a dose of 10^10^ CFU per mouse, with a volume of 200 μL. In contrast, the control group received a single gavage of 200 μL sterile saline. No further interventions were made to the mice after gavage. After the days 1, 3, and 7 gavage, six mice from each group were euthanized. Subsequently, intestinal contents were collected and stored at −80 °C (Figure 1). The Lp082 group’s small intestine was labeled as group A, and the first, third, and seventh days were named AD1, AD3, and AD7. The Lp082 group’s large intestine was labeled as group B, and the first, third, and seventh days were named BD1, BD3, and BD7. The control group’s small intestine was labeled as group C, and the first, third, and seventh days were named CD1, CD3, and CD7. The control group’s large intestine was labeled as group D, and the first, third, and seventh days were named DD1, DD3, and DD7.

### 2.3. Metagenomic Sequencing and Annotation of Gut Microbiota

The intestinal contents were thoroughly mixed and ground, and genomic DNA from the intestinal contents was extracted using the fecal DNA extraction kit (TIANGEN; #DP328-02, Tiangen Biotech Co., Ltd., Beijing, China). The purity of the DNA was measured using the NanoDrop 2000 (Thermo Fisher Scientific Co., Ltd., Beijing, China) [18] to ensure that it conformed to the measurement requirements (nucleic acid 260/280 ratios of 1.7–1.9). DNA samples were sent to Beijing Novogene Co., Ltd. (Beijing, China). for metagenomic sequencing using the Illumina Hiseq 2500 platform [19]. The raw sequencing data underwent sequential preprocessing, including adapter contamination removal and quality correction with the command “fastqc *.gz -t 4” to evaluate the average quality distribution [20]. The commands to generate a non-redundant sequence set are the following: gunzip *.gz; fq2fa --merge --filter A_1.fastq A_2.fastq A.fa; fa2fq A.fa A.fq. Microbial species and abundance annotations of the microbial metagenomic data were conducted using MetaPhlAn3 software (version 3.0) [21]. Functional gene and metabolic pathway annotations were performed using HUMAnn3 software (version 3.0.1) [22].

### 2.4. Single-Nucleotide Mutation Annotation and Statistics Analysis

The Spearman correlation between microbial species and Lp082 was calculated using the “WGCNA” package (version 1.73) [23] in R software (version 4.3.2) [24]. Species were screened based on correlation results with a *p*-value < 0.05 and correlation coefficients with Lp082 either greater than 0.5 or less than −0.5. Genomes of representative strains of these species were collected. The FASTA files of representative strain sequences with a “Reference genomes” were downloaded from the National Center for Biotechnology Information (NCBI) (Table S1), and a representative strains database was constructed using Bowtie2 [25]. The metagenomic sequencing reads from both intestinal segments at each time point were aligned against the reference database to obtain Binary Alignment/Map (BAM) files for the intestinal content samples. Mutation sites were annotated using inStrain v1.0.0 software to obtain SNV information between intestinal strains and the reference genome [26]. The command is as follows: prodigal-i/home/ref.fna-d ref_genes.fna, bowtie2-build/home/inStrain/ref.fna ref.index; bowtie2-p 36-x/home/inStrain/ref.index --no-mixed --very-sensitive --n-ceil 0,0.01-U/home/A.fq.gz-S A.sam; samtools view-bS/home/inStrain/A.sam-oA.bam; samtools sort-m 10000000000/home/inStrain/A.bam A.sorted.bam; inStrain profile/home/inStrain/A.sorted.bam/home/inStrain/ref.fna-c 100-f 0.49-o A.profile-p 36-g/home/inStrain/ref_genes.fna. Using a control group as a negative control, we excluded SNVs arising from issues related to the selection of the reference genome. The SNVs detected in both groups from the same day’s samples marked for natural variation were generated over time and subsequently removed. Ultimately, only the SNVs induced by probiotic intervention in the local gut microbiota were retained. The *.SNVs.tsv files in the output folder include details such as strain codes, mutation positions, the bases before and after the mutation, and whether the mutations are synonymous or non-synonymous, and so on.

Analysis of Variance (ANOVA) [27] was applied for comparisons among multiple groups, followed by Tukey’s test for pairwise comparisons [28]. When data did not meet the assumptions of homogeneity of variance or normal distribution, the Kruskal–Wallis test was used [29]. Statistical significance was assessed using *p* = 0.05. Utilizing the R packages “vegan” (version 2.6.8) [30], we computed the α-diversity indices Shannon and Simpson and subsequently produced boxplots (mean and SD) through the “ggplot2” package (version 3.5.0) [31]. To further verify the statistical significance of these differences, the Kruskal–Wallis test from the “permute” package (version 0.9.7) [32] was employed. β diversity based on Bray–Curtis [33] distance was calculated using the “vegan” package and subsequently visualized as a PCoA [34] plot with “ggplot2”. Box plots depicting species abundance were constructed using GraphPad Prism software (version 8.0.2) [35]. Network diagrams were then constructed using Cytoscape software (version 3.10.1) [36]. Genomic General feature format (GFF) files for the species were downloaded from NCBI to annotate gene functions. Protein prediction maps for relevant genes were generated using Protein Homology/analogY Recognition Engine V 2.0 [37].

## 3. Results

### 3.1. The Response of the Indigenous Microbiota in Different Intestinal Segments to Lp082

**Figure 2:**
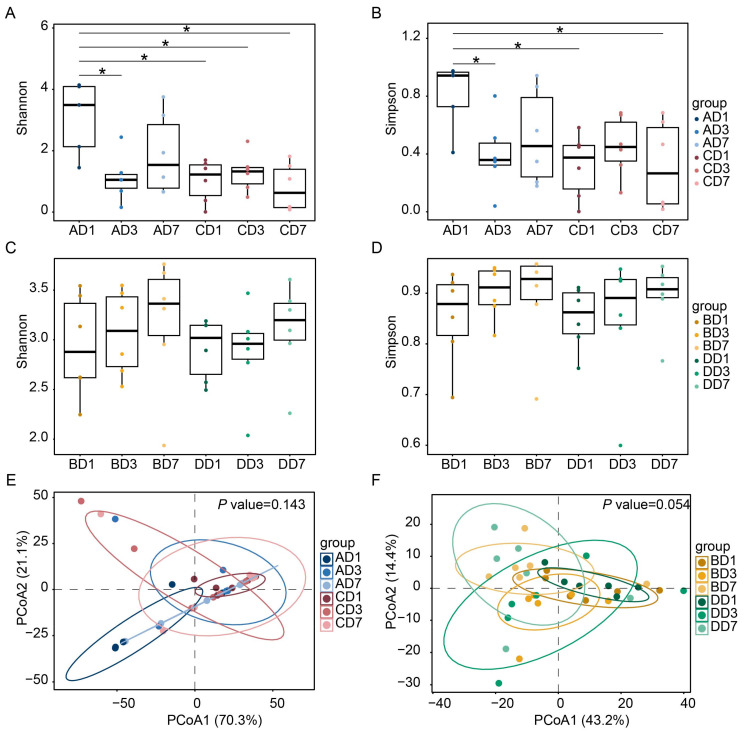
Changes in α-diversity and β-diversity of the small and large intestine. (**A**) Box plots of Shannon index for the small intestine in the Lp082 group (AD1, AD3, AD7) and control group (CD1, CD3, CD7) on days 1, 3, and 7 post-gavage. (**B**) Box plots of Shannon index for the large intestine in the Lp082 group (BD1, BD3, BD7) and control group (DD1, DD3, DD7) on days 1, 3, and 7 post-gavage. (**C**) Box plots of Simpson index for the small intestine in the Lp082 group (AD1, AD3, AD7) and control group (CD1, CD3, CD7) on days 1, 3, and 7 post-gavage. (**D**) Box plots of Simpson index for the large intestine in the Lp082 group (BD1, BD3, BD7) and control group (DD1, DD3, DD7) on days 1, 3, and 7 post-gavage. (**E**) PCoA based on Bray–Curtis distances of small intestine samples, with blue points (AD1, AD3, AD7) representing the Lp082 group and red points (CD1, CD3, CD7) representing the control group. The contribution rates of PCoA1 and PCoA2 were 70.3% and 21.1%, respectively, *p*-value = 0.143. (**F**) PCoA based on Bray–Curtis distances of large intestine samples, with yellow points (BD1, BD3, BD7) representing the Lp082 group and green points (DD1, DD3, DD7) representing the control group. The contribution rates of PCoA1 and PCoA2 were 43.2% and 14.4%, respectively, *p*-value = 0.054. Asterisks indicate statistical significance between groups in (**A**,**B**), * *p*-value < 0.05.

We annotated the abundance of gut microbiota and compared the changes in diversity of local gut microbiota in the large and small intestines following Lp082 intervention. There were no significant differences in Shannon and Simpson indices among the groups CD1, CD3, and CD7 in the small intestine. In the Lp082 group, the Shannon and Simpson indices in the small intestine significantly increased on the first day after Lp082 administration compared to the control group. This indicates a response of the microbial community in the mouse small intestine to the Lp082 intervention. However, these indices significantly decreased on the third day compared to the first day post-gavage. No significant differences were observed between the third and seventh days (Figure 2A,B). For the large intestine, there were no significant differences in Shannon and Simpson indices among the groups, nor between the control and Lp082 groups (Figure 2C,D). To assess the impact of Lp082 on microbial community structure across different intestinal segments, principal coordinates analysis (PCoA) was conducted on intestinal contents collected from both the large and small intestines at various time points. In the small intestine, no significant clustering or differences were observed between the control and Lp082 groups or among different days within each group (Figure 2E). Similarly, in the large intestine, no significant clustering or differences were evident between the control and Lp082 groups. Additionally, no significant differences were observed across days within each group (Figure 2F). These results suggested that the mouse gut microbiota exhibited stability over a seven-day period without external intervention (Lp082 in this experiment). Furthermore, the observed differences in the small intestine of the Lp082 group were specifically induced by Lp082.

### 3.2. Annotation and Identification of Lp082-Related Microbiota in Different Intestinal Segments

**Figure 3:**
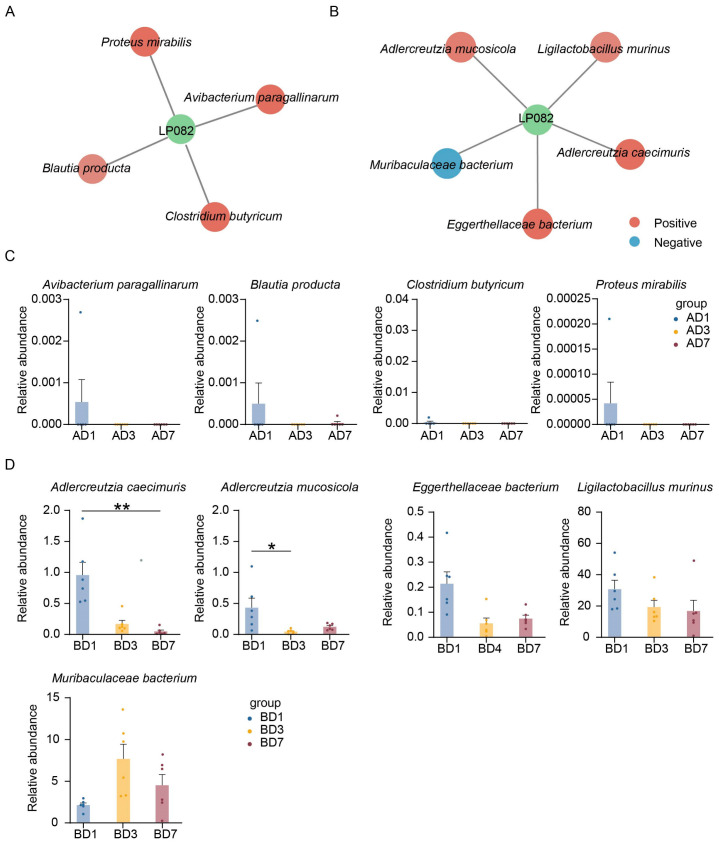
Bacterial Species and Their Abundance Responding to Lp082 Post-Gavage Administration. (**A**) Bacterial species correlated with Lp082 in the small intestine (*p*-value < 0.05, r > 0.5). (**B**) Bacterial species correlated with Lp082 in the large intestine (*p*-value < 0.05, r > 0.5, or r < −0.5). Green circles represent Lp082, red circles indicate bacteria positively correlated with Lp082, and blue circles represent bacteria negatively correlated with Lp082. The shade of color indicates the magnitude of the correlation; darker shades correspond to stronger correlations. (**C**) The abundance of bacterial species correlated with Lp082 in the small intestine at different time points. (**D**) The abundance of bacterial species correlated with Lp082 in the large intestine at different time points. Asterisks indicate statistical significance between groups in (**D**), * *p*-value < 0.05, ** *p*-value < 0.01.

The impact of Lp082 on the entire gut microbiota upon entry into the intestine was bound to manifest in individual bacterial species as well. We annotated the abundance of each bacterial species and identified those quantitatively correlated with Lp082 through Spearman correlation analysis. In the small intestine, four species positively correlated with Lp082 were identified as follows: *Avibacterium paragallinarum*, *Blautia producta*, *Clostridium butyricum*, and *Proteus mirabilis* (Figure 3A). In the large intestine, four species positively correlated with Lp082 were found as follows: *Adlercreutzia mucosicola*, *Ligilactobacillus murinus*, *Adlercreutzia caecimuris,* and *Eggerthellacea bacterium*. One species, *Muribaculaceae bacterium*, exhibited a negative correlation with Lp082 (Figure 3B). The abundance levels of individual bacterial species across the small intestine have been annotated (Figure 3C). Similarly, the abundance levels of individual bacterial species across the large intestine have been annotated (Figure 3D). The composition of microbial communities differs across different intestinal segments, and the responses of individual bacterial species to Lp082 vary accordingly.

### 3.3. Annotation of Mutation Number in the Indigenous Microbial Populations Across Different Intestinal Segments

**Figure 4:**
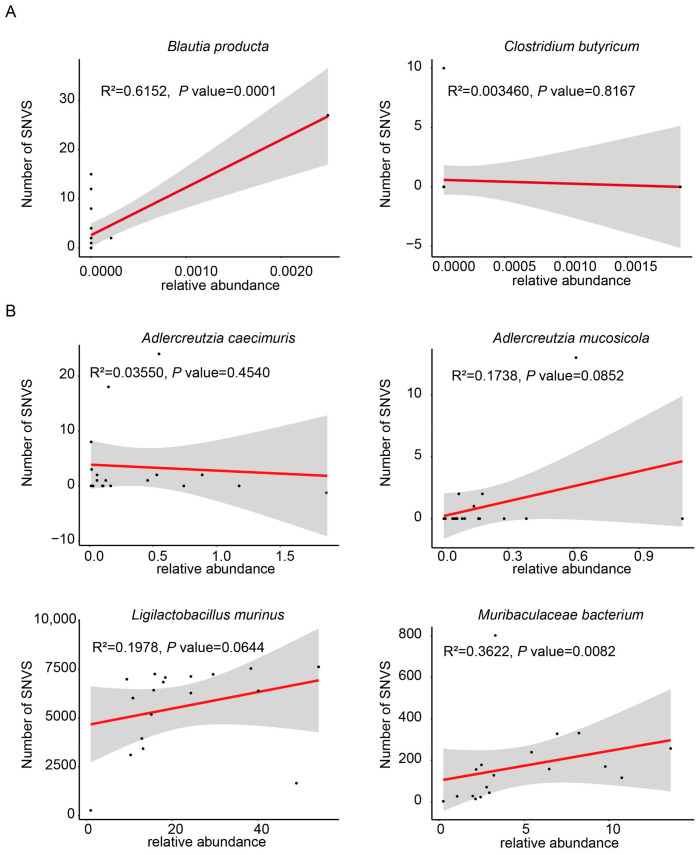
Relationship between the abundance of relevant bacterial species and the number of SNVs. (**A**) A fitted curve illustrating the relationship between the abundance of relevant bacterial species and the number of SNVs in the small intestine. (**B**) Fitted curve depicting the relationship between the abundance of relevant bacterial species and the number of SNVs in the large intestine. The black dots represent the relative abundance of bacterial species in the sample and the number of SNVs in that sample. The red lines indicate the fitted curve. The gray shaded area indicates the confidence interval (*p* value < 0.05).

**Figure 5:**
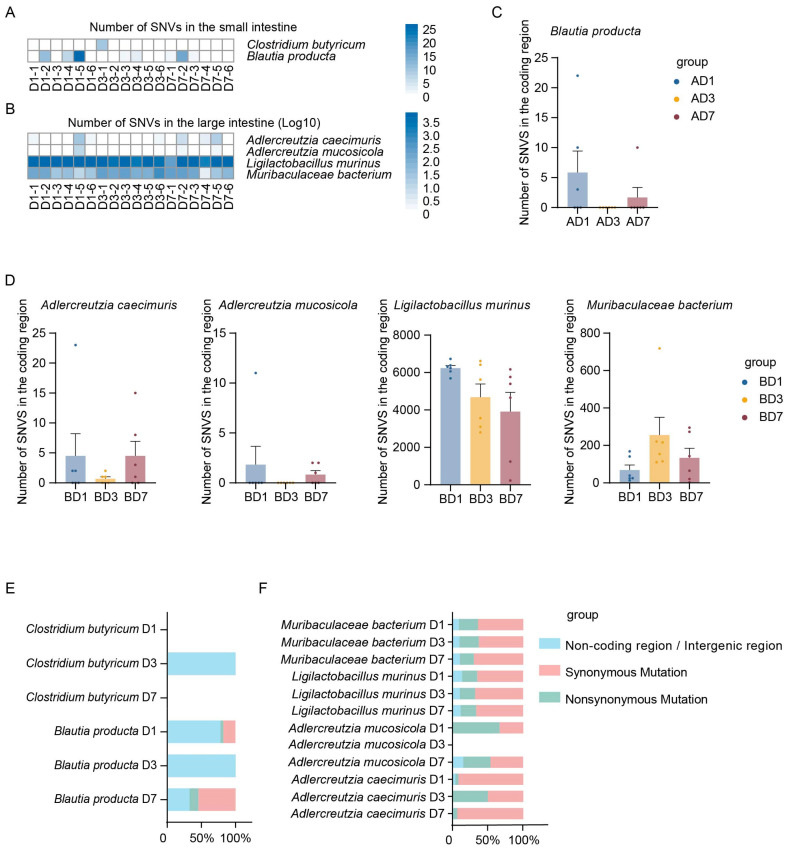
Annotation of SNVs in Relevant Bacterial Species. (**A**) The number of SNVs occurring at different time points in bacterial species correlated with Lp082 in the small intestine. (**B**) The number of SNVs occurring at different time points in bacterial species correlated with Lp082 in the large intestine. The intensity of colors in the heatmap represents the quantity of SNVs. (**C**) The number of SNVs located in coding regions at different time points in relevant bacterial species of the small intestine. (**D**) The number of SNVs located in coding regions at different time points in relevant bacterial species of the large intestine. (**E**) The proportions of SNVs located in non-coding regions, intergenic regions, and synonymous versus nonsynonymous mutations within coding regions at different time points in the small intestine. (**F**) The proportions of SNVs located in non-coding regions, intergenic regions, and synonymous versus nonsynonymous mutations within coding regions at different time points in the large intestine.

SNVs represent a common type of mutation, referring to the variation in a single nucleotide within a DNA sequence. Initially, the number of SNVs in bacterial species associated with Lp082 had been counted separately in both the large and small intestines. Next, to account for potential discrepancies between the NCBI-selected strains and the actual isolates used in this study, the mutation annotation sites were carefully examined. Specifically, the sites corresponding to the strains annotated in the control group had been removed. The relationship between the number of SNVs and bacterial abundance was analyzed, and fitting curves were plotted to represent this relationship (Figure 4A,B). No significant linear correlation was observed between these two variables. This suggests that an increase in the number of SNVs was not influenced by an increase in bacterial abundance. A heatmap was generated based on the number of SNVs in each bacterial species, with color intensity indicating the number of SNVs. In the small intestine, two species, *C. butyricum* and *B. producta*, both exhibited a relatively low number of SNVs (Figure 5A). In the large intestine, four species were annotated for the occurrence of SNVs, including *A. mucosicola*, *A. caecimuris*, *M. bacterium*, and *L. murinus. L. murinus* exhibited a significantly greater number of SNVs compared to the others (Figure 5B).

### 3.4. Annotation of Mutation Type in the Indigenous Microbial Populations Across Different Intestinal Segments

To further investigate the genomic responses of intestinal endogenous microbiota, we analyzed the types of SNVs occurring in various bacterial species. A low number of SNVs located within coding regions were observed in small intestinal bacteria, with *C. butyricum* lacking any annotated SNVs in coding regions (Figure 5C). Analysis of the types of SNVs in large intestinal bacteria revealed that *L. murinus* exhibited a significantly higher number of coding region SNVs compared to other species (Figure 5D). Regarding SNVs in coding regions, *B. producta* in the small intestine showed more synonymous mutations than nonsynonymous mutations on both the first and seventh days, with no mutations annotated on the third day. In the large intestine, *A. mucosicola* had fewer synonymous mutations than nonsynonymous mutations on the first day but more synonymous mutations on the seventh day, with no mutations annotated on the third day. *A. caecimuris* showed more synonymous mutations than nonsynonymous mutations on both the first and seventh days but fewer synonymous mutations on the third day. *M. bacterium* and *L. murinus* consistently exhibited more synonymous mutations than nonsynonymous mutations at all time points. These findings may suggest that Lp082 has a more pronounced genomic impact on *L. murinus* in the large intestine, indicating a greater response of this species to Lp082 (Figure 5E,F).

**Figure 6:**
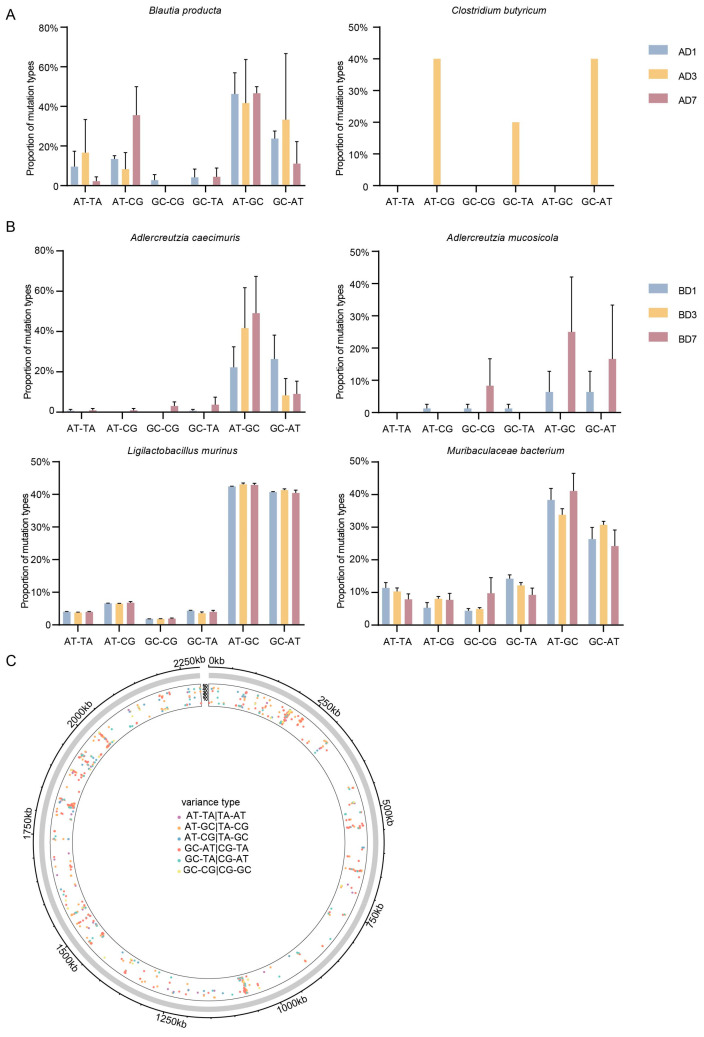
Mutation Profiles of Relevant Gut Microbial Species. (**A**) Nucleotide substitutions in SNVs of relevant microspecies in the small intestine. (**B**) Nucleotide substitutions in SNVs of relevant microspecies in the large intestine. (**C**) Sites, types, and mutation frequencies of SNVs in *L. murinus*.

Subsequently, we annotated the type of SNVs occurring in various bacterial species within the small intestine and the large intestine. The frequency of base transitions was generally high. Among the SNVs in *B. producta* in the small intestine, A-G and G-A substitutions were particularly frequent (Figure 6A). A similar pattern was also observed in *A. caecimuris*, *M. bacterium*, and *L. murinus* in the large intestine. A-G and G-A substitutions were more prevalent than other mutation types. This pattern was observed across the first, third, and seventh days of annotation. No SNVs were annotated for *A. mucosicola* on day 3; the SNVs observed on days 1 and 7 followed this method (Figure 6B).

The outermost circle indicated the genomic loci where SNVs occurred, with the total length of *L. murinus* being 2,290,452 bp. The position of the dots from the inner to the outer circle represents an increasing frequency of mutations. It was evident that SNVs in the entire genome of *L. murinus* in the large intestine were distributed across multiple positions with varying mutation frequencies (Figure 6C). The genes harboring SNVs were functionally annotated, including α-glucosidase, glycerate kinase, DMT family transporter, LytTR family DNA-binding domain, and so on. These variations could potentially impact the expression and function of multiple genes.

### 3.5. Functional Annotation of SNVs in Different Intestinal Segments

**Figure 7:**
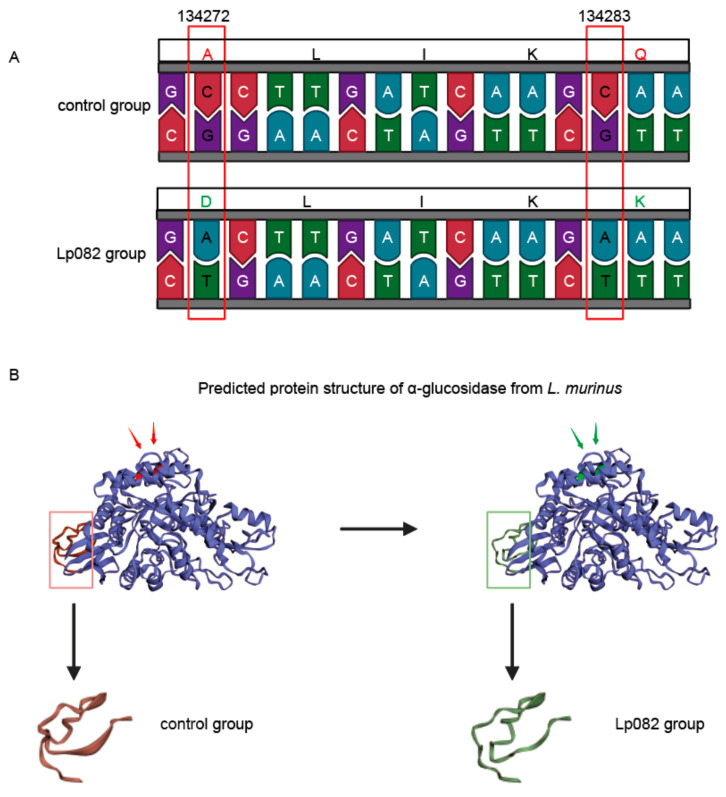
Annotation of Mutation Gene Functions. (**A**) Sites of nonsynonymous mutations in the α-glucosidase gene of *L. murinus*. Bases that have changed are highlighted by red rectangles. Amino acids that changed are shown in red font (control group) and green font (Lp082 group), respectively. (**B**) Predicted protein structure of α-glucosidase from *L. murinus*; arrows point to amino acid sites with nonsynonymous mutations. with the control group shown in red and the Lp082 group in green, revealing differences in protein structure between the two groups (highlighted by rectangles).

There exists a close relationship between genes and function, with genes capable of regulating protein function. To ascertain whether the function of a gene is affected by SNVs, the function of the genes in which SNVs are located was annotated. A large number of SNVs were found to occur on the α-glucosidase gene (WP_112195818.1) of *L. murinus* in the large intestine. Two nonsynonymous mutations (at position 134272, a single C > A mutation, and at position 134283, a single C > A mutation) were annotated in the α-glucosidase gene of *L. murinus* (Figure 7A). Specifically, the GCCTTGATCAAGCAA in the control group and GACTTGATCAAGAAA in the Lp082 group. The structure of the α-glucosidase protein was predicted, and the nonsynonymous mutations were annotated on the structural diagram. The control group is labeled red, and the Lp082 group is labeled green, revealing differences in protein structure between the two groups (highlighted by rectangles) (Figure 7B). The occurrence of non-synonymous SNVs leads to changes in protein structure, which may result in alterations in α-glucosidase function [38]. The mutations in the α-glucosidase gene of *L. murinus* in the large intestine may affect its utilization of carbohydrates.

## 4. Discussion

The microbial diversity and abundance varied across different intestinal segments in mice [39]. After entering the gut, probiotics can pass through or colonize within the intestinal tract. This process influences the abundance of the indigenous gut microbiota, promotes their evolution, and ultimately affects host health [40]. To elucidate the genomic responses of the indigenous gut microbiota to the introduction of probiotics, this study analyzed and annotated SNVs in the intestinal contents of both the small intestine and large intestine of mice after gavage with Lp082.

Our experimental results corroborated the response of microbial species, diversity, and abundance varied among different intestinal segments. Upon the gavage of Lp082, distinct responses in microbial diversity and abundance were observed in the small intestine versus the large intestine. Shannon’s and Simpson’s indices are only modified in the small intestine on the first day after strain administration, with no differences on the remaining days. This may be attributed to the self-regulating capacity of the gut microbiota, which enables it to restore homeostasis after disturbances [41]. In the large intestine, no significant differences were observed in Shannon’s and Simpson’s indices. This may be attributed to the higher abundance of microbial species in the large intestine compared to the small intestine [42]. Community ecology studies suggest that biological communities with greater abundance and diversity generally exhibit stronger ecological stability [43,44] and are less susceptible to perturbation by exogenous microorganisms [45].

The local microbial species correlated with Lp082 varied across different intestinal segments and exhibited distinct responses. In the small intestine, fewer SNVs are annotated in bacterial species, whereas in the large intestine, more SNVs are annotated in bacterial species. This may be because highly diverse communities are better able to screen for individuals or populations with stronger adaptability through natural selection when facing environmental pressures. Such communities typically exhibit greater adaptive evolutionary potential [46]. Additionally, microorganisms require a sufficient quantity and time to persist [47]. The transit time in the large intestine is around 30 h, which allows for greater diversity, richness, and abundance [48]. In contrast, the small intestine has high fluidity, with a transit time of only 3 to 5 h. At the same time, the small intestine rapidly clears and propels digestive contents downstream through mechanical actions (such as peristalsis and mixing), which also reduces the opportunity for microorganisms to colonize the intestinal wall [9]. A greater number of SNVs were annotated on *L. murinus* in the large intestine, with a higher proportion of synonymous mutations compared to nonsynonymous mutations. A higher frequency of base transitions was observed among these SNVs. Conversely, no significant change was observed in the abundance of *L. murinus* in the large intestine. For individual bacterial species, changes in abundance may be constrained by multiple complex factors. These factors include environmental fluctuations, interspecific competition, and biological adaptive adjustments. The combined effects of these factors may result in insignificant changes in species diversity and abundance levels [49]. SNVs are generally recognized as one of the core driving forces of biological evolution. Their mutations occur randomly and typically precede shifts in species abundance in natural environments [50,51]. For instance, mutations in genes associated with species immunity have led to the emergence of a highly resistant population within a particular species. This resistance has enabled their survival and subsequently resulted in an increase in the species’ abundance [52]. It suggests a greater emphasis should be placed on the genomic responses of the indigenous microbiota following the introduction of probiotic Lp082 into the intestine.

The occurrence of SNVs may impact the gene functions and metabolism of the bacterial species. And certain SNVs can help gut microbiota adapt to environmental changes within the host (such as pH, nutrient conditions, antibiotics, etc.), serving as the foundation for microbial genome evolution [53]. The occurrence of some SNVs can influence the expression of gene functions [51,54]. SNVs can also serve as a method for assessing gut health [55]. The specific gene prediction models established based on the analysis of gut microbial SNVs demonstrate high accuracy in detecting Crohn’s disease [56].

SNVs caused by reference genome selection and natural variations over time were excluded, and the occurrence of the remaining SNVs was attributed to the intervention of Lp082. The functions of the genes where SNVs were located were annotated, such as α-glucosidase, glycerate kinase, DMT family transporter, and LytTR family DNA-binding domain. Variations in these genes may affect the physiological processes of *L. murinus*. A substantial number of SNVs were observed in the α-glucosidase gene in *L. murinus* within the large intestine. The α-glucosidase gene is a common gene involved in carbohydrate utilization [57]. Research indicates that the probiotic strain Lp082 encounters intense competition with indigenous gut species owing to shared carbohydrate utilization profiles. Furthermore, Lp082 exerts a notable impact on the carbohydrate-active enzymes of the resident gut microbiota [58]. Thereby exerting an impact on the ecological and genetic stability of the gut microbiota, further influencing the structure of the intestinal microbiota. Meanwhile, LP082 promotes the growth of beneficial bacteria by metabolizing carbohydrates to produce short-chain fatty acids, among other metabolites [17,59].

However, this study still has some limitations. It is currently generally believed that the response of the gut microbiome to probiotics at the genomic level occurs prior to changes in abundance and other functional alterations [51,55,56]. This study examined the effects of probiotic intervention on the gut microbiome using a single administration. The results revealed the occurrence of microbial genomic mutations within a short-term timeframe. Future research should further explore how the gut microbiome genetically adapts under long-term probiotic administration. In this study, the genetic-level responses of native gut microbiota were investigated through metagenomic data analysis. SNVs in α-glucosidase genes within the large intestine of *L. murinus* were annotated. In future experiments, the effect of SNVs in α-glucosidase genes on metabolic processes will be evaluated. *L. murinus* strains isolated from the large intestine will be used to study their carbohydrate utilization capacity. The research will be conducted based on in vivo and in vitro experiments. Combined with metabolomics, transcriptomics, and genomics, to better understand the physiological implications of SNVs on α-glucosidase and how these changes affect host health.

## 5. Conclusions

In summary, our segmented intestinal analysis indicated that the indigenous gut microbiota exhibited differential responses to exogenous probiotics after probiotic ingestion in mice. To acquire essential nutrients and other resources for survival, some microbial species underwent evolution. *L. murinus* may alter its carbohydrate utilization through mutations in its α-glucosidase gene. These findings reveal that probiotics not only influence the diversity and abundance of the indigenous gut microbiota but also promote their evolution. This study investigates the evolutionary effects of probiotics on gut microbiota. It offers new insights into probiotic mechanisms and establishes a scientific basis for developing more precise and effective intervention strategies.

## Data Availability

All the metagenomic sequencing data reported in this paper have been deposited in the NCBI database under project number PRJNA1035164.

## Supplementary Materials

The following supporting information can be downloaded at https://www.mdpi.com/article/10.3390/microorganisms13040731/s1, Table S1: Representative strains information.
