# Supplementary material for: Distinct Effects of Lactiplantibacillus plantarum HNU082 on Microbial Single-Nucleotide Variants in Large Intestine and Small Intestine
Source: Microorganisms. 2025 Mar 25;13(4):731. doi: 10.3390/microorganisms13040731 (PMC12029867; doi:10.3390/microorganisms13040731)
Supplement: Supplementary file 1 [file microorganisms-13-00731-s001.zip › microorganisms-3524565-supplementary.pdf]

Table S1: Representative strains information

| Strains | Assembly | GenBank | Accession Number | Strain | Genome size |
|---|---|---|---|---|---|
| *Avibacterium_paragallinarum* | ASM1176560v1 | GCA_011765605.1 | NZ_CP050316.1 | ESV-135 | 2.5 Mb |
| *Blautia_producta* | ASM1066920v1 | GCA_010669205.1 | NZ_CP048626.1 | JCM 1471 | 6.2 Mb |
| *Clostridium_butyricum* | ASM188687v1 | GCA_001886875.1 | NZ_CP013239.1 | CDC_51208 | 4.6 Mb |
| *Proteus_mirabilis* | ASM6996v1 | GCA_000069965.1 | NC_010554.1 | HI4320 | 4.1 Mb |
| *Adlercreutzia_mucosicola* | ASM42262v1 | GCA_000422625.1 | NZ_KE383895.1 | AUGK01 | 3 Mb |
| *Ligilactobacillus_murinus* | ASM328811v1 | GCA_003288115.1 | NZ_CP023565.1 | CR147 | 2.3 Mb |
| *Eggerthellaceae_bacterium* | UHGG_MGYG-HGUT-02442 | GCA_902387185.1 | NZ_CABMLF010000001.1 | / | 3.9 Mb |
| *Adlercreutzia_caecimuris* | Ente_caec_B7_V1 | GCA_000403355.2 | NZ_KE159646.1 | B7 | 3 Mb |
| *Muribaculaceae_bacterium* | Strain_X | GCA_951635545.1 | OX620685.1 | Strain_X | 3.6 Mb |
